# The efficacy and safety of moxibustion for pressure injury: A protocol for systematic review and meta-analysis

**DOI:** 10.1097/MD.0000000000028734

**Published:** 2022-02-11

**Authors:** Wei Xiang, Jianmei Jiang, Tingting Hu, Xiaoling Deng, Cheng Chen, Zhongrong Chen

**Affiliations:** The Central Hospital of Enshi Tujia and Miao Autonomous Prefecture, Enshi, Hubei Province, China.

**Keywords:** meta-analysis, moxibustion, pressure injury, protocol, systematic review

## Abstract

**Background:**

Pressure injury is an important global health issue characterized by the high incidence, rapid progression, and difficult healing. How to perform timely treatment and care have been the current focus and challenge for health care professionals. Moxibustion can improve skin microcirculation, promote blood circulation, activate tissue cells, inhibit, and kill bacteria on the wounded surface, thus promoting wound healing. However, the clinically reported efficacy of moxibustion in the treatment of pressure injuries varies a lot and lacks evidence-based medical evidence. Therefore, this meta-analysis aims to evaluate the efficacy and safety of moxibustion on the treatment of pressure injuries.

**Methods:**

Randomized controlled trials (RCTs) reporting the moxibustion for pressure injury published before January 2022 will be searched in online databases, including the Chinese Scientific Journal Database, China National Knowledge Infrastructure Database, Wanfang Database, China Biomedical Literature Database, PubMed, Cochrane Library, Embase, and Web of Science. References of eligible literatures will be manually reviewed. According to inclusion and exclusion criteria, literature screening, data extraction and quality assessment will be independently performed by 2 reviewers, and meta-analysis of relevant data will be conducted using Stata14.0 software.

**Results:**

The study will provide a high-quality convincing assessment of efficacy and safety of moxibustion for pressure injury.

**Conclusion:**

The results of this study will provide the latest evidence support for judging the efficacy and safety of moxibustion on the treatment of pressure injury.

**OSF Registration number:**

DOI 10.17605/OSF.IO/T543Y.

## 1 Introduction

Pressure injury is a surgical disorder resulting in tissue ulceration and necrosis due to long-term local tissue pressure followed by persistent ischemia, hypoxia, and malnutrition.^[1–3]^ Modern medicine considers pressure injuries as chronic, difficult-to-heal wounds, which are characterized by a lack of microvascular count and repair factors, tissue hypoxemia, and delayed epithelialization.^[4]^ Would healing will be difficult following pressure injuries.^[5]^ Currently, the treatment of pressure injuries has a poor outcome with high medical cost.^[6]^ Therefore, searching for safe and effective therapeutic strategies with a low cost has become the focus of medical research at home and abroad.

In traditional Chinese medicine (TCM), pressure sores are considered as the syndrome of deficiency and stagnation of Qi and blood due to the deficiency in the body from prolonged illness or injury from long-term lie in bed, resulting in localized flesh rot and necrosis caused by the loss of moistening of the skin and toxic decay.^[7]^ Moxibustion has the effect of moving Qi, activating blood circulation and eliminating stasis.^[8]^ The heat stimulation can expand the local capillaries to make them congested and accelerate the local microcirculatory flow, thus affecting the metabolism of the tissue fluid in the whole body.^[9,10]^ Moreover, moxibustion stimulates cellular vitality, strengthens the body's ability to resist injury and antioxidation, and improves the immunity.^[11,12]^ The warming effect of moxibustion can support the body's positive energy and enhance the driving force of the meridians, recovering the normal transition of the meridians and blood.^[13]^ Moxibustion has been used in the treatment of various diseases, such as facial nerve palsy, chronic fatigue, osteoarthritis of the knee and type 2 diabetes, pressure sores and other chronic wound care.^[14–19]^

In recent years, a growing number of clinical studies have shown the therapeutic efficacy of moxibustion on the pressure injury.^[13,20–24]^ However, its efficacy and safety on the treatment of pressure injury have not been systematically evaluated. Therefore, this study aims to evaluate the efficacy and safety of moxibustion on the treatment of pressure injury by meta-analysis.

## 2 Methods

### 2.1 Protocol

Under the guidance of the Preferred Reporting Items for Systematic Reviews and Meta-Analysis Protocols (PRISMA-P), this protocol of systematic review and meta-analysis has been drafted.^[25]^ The research framework has been registered on the open science framework (Registration Number: DOI 10.17605/OSF.IO/T543Y).

### 2.2 Ethics

Since this is a protocol without patient recruitment and personal information collection, the approval of the ethics committee is not required.

### 2.3 Eligibility criteria

#### 2.3.1 Types of studies

All randomized controlled trials (RCTs) of moxibustion for the treatment of pressure injuries published in Chinese and English will be included.

#### 2.3.2 Types of participants

Patients with pressure ulcers older than 18 years.

#### 2.3.3 Types of interventions

Regular turning over, air bed and other basic treatment are given in both experimental group and control group. Moxibustion therapy or moxibustion combined with other therapies is additionally given to patients with pressure ulcers in experimental group. Conventional dressing change or other combined placebo treatment is given to those in control group.

#### 2.3.4 Types of outcome measurements

Total effective rate, cure rate, healing time, and adverse events.

### 2.4 Exclusion criteria

1. Animal experiments;
2. Studies with incomplete data;
3. Repeatedly published literatures;
4. Reviews, techniques, case reports, letters to the editor, and editorials.

### 2.5 Searching strategy

We will systematically search relevant RCTs of moxibustion for the treatment of pressure injuries published before January 2022 in the following databases: Chinese Scientific Journal Database, China National Knowledge Infrastructure Database, Wanfang Database, China Biomedical Literature Database, PubMed, Cochrane Library, Embase, and Web of Science. Searching strategy in Pubmed was shown in Table 1. Modified search strategies will be adopted during literature search in other electronic databases.

**Table 1:** PubMed search strategy.

Number	Search terms
#1	Pressure Ulcer [MeSH]
#2	Bedsore [Title/Abstract]
#3	Decubitus Ulcer [Title/Abstract]
#4	Pressure Sore [Title/Abstract]
#5	Bed Sores[Title/Abstract]
#6	Bed Sore [Title/Abstract]
#7	Bedsores[Title/Abstract]
#8	Decubitus Ulcers[Title/Abstract]
#9	Pressure Sores [Title/Abstract]
#10	Pressure Ulcers [Title/Abstract]
#11	Sore, Bed [Title/Abstract]
#12	Sore, Pressure [Title/Abstract]
#13	Sores, Bed [Title/Abstract]
#14	Sores, Pressure [Title/Abstract]
#15	Ulcer, Decubitus [Title/Abstract]
#16	Ulcer, Pressure [Title/Abstract]
#17	Ulcers, Decubitus [Title/Abstract]
#18	Ulcers, Pressure [Title/Abstract]
#19	Pressure injury [Title/Abstract]
#20	or/1-19
#21	Moxibustion [MeSH]
#22	Moxibustion [Title/Abstract]
#24	or/21-22
#24	Randomized Controlled Trials as Topic [MeSH]
#25	Clinical Trials, Randomized[Title/Abstract]
#26	Controlled Clinical Trials, Randomized[Title/Abstract]
#27	Trials, Randomized Clinical[Title/Abstract]
#28	Random^∗^[Title/Abstract]
#29	or/24-28
#30	#20 and #24 and #29

### 2.6 Data screening and extraction

Data extraction after reading the full-text will be independently performed by 2 researchers according to the inclusion and exclusion criteria, which will be cross-checked. Any disagreement will be solved by the discussion with the third reviewer. The following data will be extracted:

1. the authors, year, title, and journal of the included studies;
2. baseline characteristics of patients in both groups like the sample size, sex ratio, and age, and the specific interventions and duration of interventions of the 2 groups;
3. the type of pressure injury, pressure injury staging, number of pressure injury, and pressure injury area;
4. the outcome indicators and the assessment results of the outcome indicators of the included studies; and
5. the detail for evaluating the quality of the literature.

**Figure 1:**
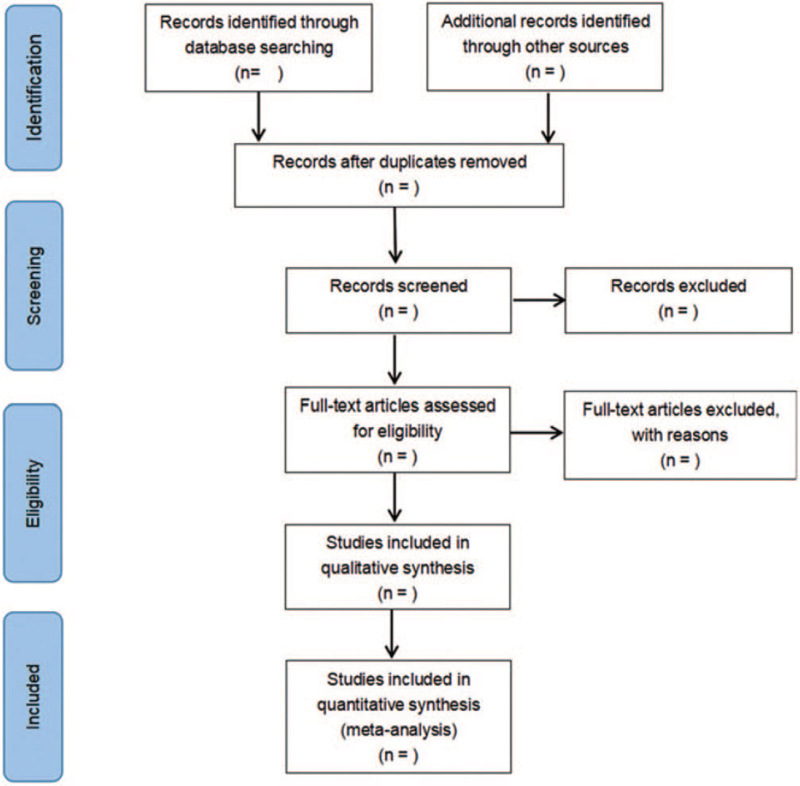
Flow diagram of literature retrieval.

The detailed selection process will be presented as a PRISMA flowchart (Fig. 1).

### 2.7 Quality evaluation

Two reviewers will independently assess the risk of bias of included RCTs using the Cochrane Systematic evaluation Handbook,^[26]^ and any disagreement will be resolved by discussing with a third reviewer.

### 2.8 Statistical analysis

Meta-analysis will be performed using the Stata 14.0 (StataCorp LLC, college station, TX) software. Relative risk with 95% confidence interval (CI) will be calculated to measure the curative effect for binary variables, and the standardized mean difference (SMD) with corresponding 95% CI will be used for continuous data. Heterogeneity between studies will be assessed by *I*-square (*I*^*2*^) and *Q*-statistic (*P* < .10), and *I*^*2*^ > 50% will be recognized as heterogeneity.^[27]^ If *P* ≥ 0.1 and *I*^*2*^≤ 50%, a fixed-effect model (Mantel–Haenszel method) will be adopted for analysis; Otherwise, a random-effect model will be used.

#### 2.8.1 Dealing with missing data

Insufficient or missing data in the literature will be obtained by e-mailing the authors. If data are still not available, only the current available data will be analyzed and the potential impacts will be discussed.

#### 2.8.2 Subgroup analysis

Subgroup analysis based on the treatment cycle, time of each moxibustion treatment, and means of intervention if a significant heterogeneity exists.

#### 2.8.3 Sensitivity analysis

The purpose of sensitivity analysis is to determine the sources of heterogeneity and confounders and, if the trial data are sufficient, low-quality or high-quality studies will be removed one by one for sensitivity analysis of the remaining.

#### 2.8.4 Publication bias

If the number of included studies is no less than 10, a funnel plot will be drawn to assess publication bias.^[28,29]^

## 3 Discussion

Pressure injury is a common clinical care problem and nursing challenge.^[30]^ The TCM theory believes that pressure injuries are mostly caused by Qi deficiency, and blood stasis.^[31]^ Moxibustion is able to activate blood stasis, warm the meridians, and clear the channels.^[32,33]^ Therefore, moxibustion is one of the most important treatments for pressure injury. Modern medical studies have shown that moxibustion can increase the mean blood perfusion and promote wound healing, which is consistent with the theory of activating blood circulation and resolving blood stasis in TCM.^[34–36]^ Moxibustion, as a TCM nursing technique that can be independently operated by nursing staff, can alleviate the medical burden of patients to a certain extent due to the low cost of moxibustion materials.^[7]^ Therefore, moxibustion is easily performed and accepted in the treatment and care of stress injuries, serving as a better choice for clinical treatment of stress injuries. This systematic review and meta-analysis will summarize the latest RCTs to determine the efficacy and safety of moxibustion on the treatment of pressure injuries. We hope that this study will provide a basis for the treatment of pressure injury with moxibustion.

## Author contributions

**Conceptualization:** Zhongrong Chen, Wei Xiang.

**Data curation:** Wei Xiang, Jianmei Jiang.

**Formal analysis:** Jianmei Jiang.

**Funding acquisition:** Zhongrong Chen.

**Investigation:** Jianmei Jiang.

**Methodology:** Jianmei Jiang, Tingting Hu.

**Project administration:** Zhongrong Chen.

**Resources:** Tingting Hu, Xiaoling Deng.

**Software:** Tingting Hu, Xiaoling Deng.

**Supervision:** Zhongrong Chen.

**Validation:** Xiaoling Deng, Cheng Chen.

**Visualization:** Xiaoling Deng, Cheng Chen.

**Writing – original draft:** Zhongrong Chen, Wei Xiang.

**Writing – review & editing:** Zhongrong Chen, Wei Xiang.
